# Prevalence of mutations in the cysteine desulfurase IscS (*Pfnfs1*) gene in recurrent *Plasmodium falciparum* infections following artemether-lumefantrine (AL) and dihydroartemisinin-piperaquine (DP) treatment in Matayos, Western Kenya

**DOI:** 10.1186/s12936-023-04587-2

**Published:** 2023-05-19

**Authors:** Beatrice Gachie, Kelvin Thiong’o, Brenda Muriithi, Jean Chepngetich, Noah Onchieku, Jeremiah Gathirwa, Peter Mwitari, Gabriel Magoma, Daniel Kiboi, Francis Kimani

**Affiliations:** 1 Department of Molecular Biology and Biotechnology, Pan African University Institute for Basic Sciences, Technology and Innovation, P.O. Box 62000-00200, Nairobi, Kenya; 2 Centre for Traditional Medicine and Drug Research (CTMDR), Kenya Medical Research Institute, Off Raila Odinga Way, P.O. Box 54840-00200, Nairobi, Kenya; 3 Centre for Biotechnology Research and Development (CBRD), Kenya Medical Research Institute, Off Raila Odinga Way, P.O. Box 54840-00200, Nairobi, Kenya; 4 Department of Biochemistry, Jomo Kenyatta University of Agriculture and Technology (JKUAT), P.O. Box 62000 -00200, Nairobi, Kenya

**Keywords:** *Plasmodium falciparum*, Cysteine desulfurase, Artemether-lumefantrine, Dihydroartemisinin-piperaquine, Recurrent infections

## Abstract

**Background:**

Malaria remains a public health concern globally. Resistance to anti-malarial drugs has consistently threatened the gains in controlling the malaria parasites. Currently, artemether-lumefantrine (AL) and dihydroartemisinin-piperaquine (DP) are the treatment regimens against *Plasmodium falciparum* infections in many African countries, including Kenya. Recurrent infections have been reported in patients treated with AL or DP, suggesting the possibility of reinfection or parasite recrudescence associated with the development of resistance against the two therapies. The *Plasmodium falciparum* cysteine desulfurase IscS (*Pfnfs1*) K65 selection marker has previously been associated with decreased lumefantrine susceptibility. This study evaluated the frequency of the *Pfnfs1* K65 resistance marker and associated K65Q resistant allele in recurrent infections collected from *P. falciparum*-infected individuals living in Matayos, Busia County, in western Kenya.

**Methods:**

Archived dried blood spots (DBS) of patients with recurrent malaria infection on clinical follow-up days after treatment with either AL or DP were used in the study. After extraction of genomic DNA, PCR amplification and sequencing analysis were employed to determine the frequencies of the *Pfnfs1* K65 resistance marker and K65Q mutant allele in the recurrent infections. *Plasmodium falciparum msp1* and *P. falciparum msp2* genetic markers were used to distinguish recrudescent infections from new infections.

**Results:**

The K65 wild-type allele was detected at a frequency of 41% while the K65Q mutant allele was detected at a frequency of 22% in the recurrent samples. 58% of the samples containing the K65 wild-type allele were AL treated samples and while 42% were DP treated samples. 79% of the samples with the K65Q mutation were AL treated samples and 21% were DP treated samples. The K65 wild-type allele was detected in three recrudescent infections (100%) identified from the AL treated samples. The K65 wild-type allele was detected in two recrudescent DP treated samples (67%) while the K65Q mutant allele was identified in one DP treated (33%) recrudescent sample.

**Conclusions:**

The data demonstrate a higher frequency of the K65 resistance marker in patients with recurrent infection during the study period. The study underscores the need for consistent monitoring of molecular markers of resistance in regions of high malaria transmission.

**Supplementary Information:**

The online version contains supplementary material available at 10.1186/s12936-023-04587-2.

## Background

Globally, malaria remains a disease of significant public health concern. In 2020, 627,000 deaths and 241 million cases of the disease were reported [1]. Malaria is caused by an apicomplexan parasite of the *Plasmodium* genus. The deadliest species, *Plasmodium falciparum*, is the most prevalent species on the African continent, where 95% of total malaria cases occur [1].

Artemisinin-based combination therapy (ACT) is used to treat *P. falciparum* malaria due to the efficacy of combining a short-acting artemisinin derivative with a longer half-life partner drug that eradicates residual parasite load [2]. The emergence of artemisinin-resistant *P. falciparum* parasites was first reported in South-East Asia [3]. ACT remains effective in most parts of Africa [4–6]. However, reports of artemisinin-resistant parasites associated with delayed parasite clearance have been reported in Rwanda [7] and Uganda [8]. The loss of artemisinin efficacy will likely put selection pressure and affect the activity of ACT partner drugs [9]. An additional 78 million malaria cases would be reported in Africa if artemisinin and partner drug resistance develop [10]. Anti-malarial drug resistance is monitored by in vitro drug sensitivity tests, in vivo therapeutic efficacy studies, or surveillance of molecular markers associated with resistance. A possible outcome of anti-malarial drug therapeutic efficacy studies is recurrent infections during follow-up days attributed to reinfection or parasite recrudescence.

Artemisinin resistance is mainly associated with mutations in several loci of the *P. falciparum* kelch13 (*PfK13*) gene [11–13]. Resistance to the 4-aminoquinoline partner drugs, such as amodiaquine, develops through mutations in the *P. falciparum* chloroquine resistance transporter (PfCRT) and the *P. falciparum* multidrug resistance 1 transporter (PfMDR1) [13], while that of piperaquine is through plasmepsin II and III amplification [14]. The mechanism of action of aryl-alcohol drugs, such as lumefantrine is not well understood, though they are widely thought to interfere with the haem detoxification [15]. Resistance to lumefantrine has been associated with an increase in the copy number of the *pfmdr1* gene [16]. Studies on parasite resistance to lumefantrine are challenging due to poor drug solubility and the lack of a clearly defined in vitro threshold for lumefantrine resistance [17]. Therefore, monitoring molecular markers associated with parasite resistance to artemisinin and its partner drugs is necessary for early detection and response to emerging drug resistance.

In Kenya, 70% of the entire population is at risk of contracting malaria [18]. Approximately 19% of outpatient health facility visits in the country are attributed to the disease [19]. ACT was adopted as a treatment for malaria in the country in 2004. Artemether-lumefantrine (AL) is the first-line drug combination, while dihydroartemisinin-piperaquine (DP) is the second-line for uncomplicated falciparum malaria [20]. ACT remains effective against *P. falciparum* infections in Kenya [21]. Non-synonymous mutations in the PfK13 propeller region have been identified in the country, but none are associated with artemisinin resistance [22, 23].

*Plasmodium falciparum* cysteine desulfurase IscS (*Pfnfs1*) (PF3D7_0727200) has been associated with lumefantrine resistance. In the Gambia, higher IC_50_ values for the wild-type K65 alleles and a strong temporal differentiation of the same allele over seven years were associated with the lumefantrine resistance selection [24]. The PfNFS1 protein belongs to a group of transferase enzymes known as cysteine desulfurases. They are essential to Fe-S biogenesis pathways, where they are involved in the cleavage of sulfur atoms from L-cysteine for the assembly of iron-sulfur (Fe-S) clusters. FeS cluster assembly incorporates several components, including iron and sulfur sources, scaffold proteins, carrier proteins, and accessory proteins [25]. These clusters activate proteins involved in gene expression, cell development, drug resistance, and metabolic regulation during stress conditions [26–28]. The PfNFS1 protein is part of the iron-sulfur cluster synthesis (ISCS) pathway that is localized within the parasitic mitochondria [29], and the pathway is involved in asexual blood-stage parasite development as well as a possible drug target [26]. Given the pivotal role of iron homeostasis in malaria parasite blood stages and drug mechanisms of the quinolines [24], genes such as *Pfnfs1* need further studies to determine their potential roles in drug action and resistance.

This study analysed archived DBS collected on days 21, 28, and 42 post-AL and DP treatment to determine the frequency of mutations in the target region of the *Pfnfs1* gene and associated outcomes in recurrent infections. To investigate mutations in the target region of the *Pfnfs1* gene, DNA was extracted from the DBS, followed by PCR amplification and sequencing of the isolates.

## Methods

### Study site

The study was carried out in Matayos, Busia County, Western Kenya. Matayos is located at a latitude of 0.3618°N and longitude of 34.168°E, lies at an altitude is 1214 m above sea level, and has an area of 196.2km^2^. The region experiences two rainy seasons annually, from March to June and October to November. The area has a high malaria transmission and is part of the Lake Victoria basin, thereby providing suitable breeding conditions for the main malaria vectors, *Anopheles gambiae,* and *Anopheles funestus,* which facilitate continuous malaria transmission, while *P. falciparum* is the most prevalent parasite species in the area [30].

### Sample selection

Archived DBS collected in 2016 from a two-arm therapeutic efficacy study (TES) on artemether-lumefantrine (AL), and dihydroartemisinin-piperaquine (DP) were used in this study. A randomized controlled study design was employed where participants were monitored during treatment for adherence to the treatment guidelines. The treatment follow up days were day 7, 14, 21, 28 and 42 post-treatment. The study was conducted without bias towards either sex or gender. The archived DBS were stored at − 20 °C. The study's eligibility criteria included: obtaining informed consent, a history of fever with a body temperature of ≥ 37.5 °C, *P. falciparum* mono-infection, and parasitaemia levels of between 2000 and 200,000 parasites/μL of blood. The exclusion criteria included patients treated for malaria in the preceding fourteen days and voluntary withdrawals from the study. Malaria infection was confirmed using microscopy. The study analysed 71 DBS from patients with recurrent malaria infections. These DBS were collected from days 21, 28, 42, and 5 samples from unscheduled days post-drug treatment with either AL or DP.

### Distinguishing recrudescent and new infections

Two highly polymorphic markers, *P. falciparum msp2* (*Pfmsp2)* and *P. falciparum msp1* (*Pfmsp*1) were utilized to differentiate recrudescent infections from new infection following the standard methods [31]. The *Pfmsp2* gene was amplified using the forward primer 5′-GAAGGTAATTAAAACATTGTC-3′ and reverse primer 5′-GAGGGATGTTGCTGCTCCACA-3 for the primary PCR and forward primer 5′-GAGTATAAG GAGAAGTATG-3′ and reverse primer 5′-CTAGAACCATGCATATGTCC-3′ for the secondary PCR. The *Pfmsp1* gene was amplified using the forward primer 5ʹ-CTAGAAGCTTTAGAAGATGCAGTATT-3ʹ and reverse primer 5ʹ-CTTAAATAGTATTCTAATTCAAGTGGATCA-3 for the primary PCR and forward primer 5-AAATGAAGAAGAAATTACTACAAAAGG-3ʹ and reverse primer 5ʹ-GCTTGCATCAGCTGGAGGGCTTGCACC-3ʹ for the secondary PCR. Both the *Pfmsp2* and *Pfmsp1* PCR reactions were conducted under similar conditions. Briefly, the reaction mixture consisted of 5 × Firepol mastermix with 12.5 mM MgCl_2_ (Solis Biodyne™, Cat No 04-12-00125), 10 µM of both the forward and reverse primers, nuclease-free water and 1 µl of individual genomic DNA extract to a final reaction volume of 20 µl. The PCR amplification was carried out in the ProFlex PCR system (Applied Biosystems) using the following optimized conditions: Primary PCR: initial denaturation at 94 °C for 3 min, followed by 30 cycles of denaturation at 94 °C for 25 s, annealing at 42 °C for 1 min and elongation at 65 °C for 2 min followed by a final extension of 72 °C for 3 min. Secondary PCR: initial denaturation at 94 °C for 3 min, followed by 30 cycles of denaturation at 94 °C for 25 s annealing at 50 °C for 1 min and elongation at 72 °C for 3 min followed by a final extension of 72 °C for 2 min.

### DNA extraction, PCR amplification, and sequencing of the target region of the *Pfnfs1* gene

Analysis of *Pfnfs1* was performed using archived DBS from patients with recurrent parasitaemia. Parasite genomic DNA was extracted from the DBS using the QIAamp DNA mini kit (Qiagen, Cat No 51304) following instructions from the manufacturer. The target region of the *Pfnfs1* gene was amplified by conventional PCR using the forward primer 5’- TTTGTGTTAAAAGACCTCATCCC-3’ and reverse primer 5’- TCTTGGGTCAATCATTGTGGTT-3’ designed using Benchling. Briefly, the reaction mixture consisted of 5 × Q5 reaction buffer (NEB™, Cat No B9027S), 10 µM of both the forward and reverse primers, 10 mM dNTPs mix, Q5 high-fidelity DNA polymerase (NEB, Cat No M0491), nuclease-free water and 1 µl of individual genomic DNA extract to a final reaction volume of 25 µl. The PCR amplification was carried out in the ProFlex PCR system (Applied Biosystems) using the following optimized conditions: initial denaturation at 98 °C for 30 s, followed by 35 cycles of denaturation at 98 °C for 15 s, annealing at 58 °C for 15 s and elongation at 72 °C for 2 min followed by a final extension of 72 °C for 7 min. The PCR products were analysed in a 1.5% agarose gel and purified using the QIAquick PCR purification kit (Qiagen, Cat No 28104) as per manufacturer instructions. The purified PCR samples were sequenced using a 3730xl DNA Analyzer sequencer BigDye v3.1 (Applied Biosystems).

### Statistical analysis

Molecular Evolutionary Genetics Analysis (MEGA) software version 10 [32] was used to analyse the sequences with the reference gene from the genome of the 3D7 *P. falciparum* strain obtained from PlasmoDB. Frequencies were calculated as the percentage of sequences that carried a mutation out of the total number of sequences with data for the locus of interest.

### Ethical approval.

The study was conducted at the Kenya Medical Research Institute (KEMRI). All experiments were carried out as per relevant national and international standards and approved by the Scientific and Ethics Review Unit (SERU) of the Kenya Medical Research Institute under approval KEMRI/SERU/CTMDR/095/4172. The original study was a therapeutic efficacy study on the efficacy of ACT and was under SERU approval number SSC 2276.

## Results

### Baseline clinical data

A total of 334 patients were recruited into the study; 166 patients were treated with AL and 168 with DP. The median age of patients in the AL treatment arm was 56 months, while that in the DP arm was 48 months. 46% of patients in the AL treatment arm and 45% in the DP treatment arm were female. A total of 71 patients with recurrent parasitaemia were recorded at different follow-up time points. Recurrent infection following AL or DP treatment was recorded on days 21, 28, and 42 and unscheduled days post-treatment. The target region of the *Pfnfs1* gene was successfully amplified and sequenced in 64 samples: 44 AL-treated patients and 20-DP-treated patients. 26 AL-treated samples and 14 DP-treated samples sequenced were confidently scored. 24 samples (18 AL-treated and 6 DP-treated samples) were not confidently scored and thus not included for further analysis. On day 21 post-treatment, 13 AL-treated patients had recurrent parasitaemia, while DP-treated patients had no recurrent infection. On day 28 post-treatment, 20 patients exhibited recurrent infection: 17 from the AL-treated and three from DP-treated patients. Further follow-up revealed that 12 AL-treated and 14 DP-treated patients had recurrent parasitaemia yielding a total of 26 patients on day 42 post-treatment. Two AL-treated and 3 DP-treated samples showed recurrent parasitaemia on unscheduled treatment follow-up days (Table 1).

**Table 1:** A summary of recurrent infections on day 21, day 28, day 42, and unscheduled days following treatment with artemether-lumefantrine (AL) or dihydroartemisinin-piperaquine (DP)

DRUG	Post treatment day	No of recurrent infections
AL	D21	13
	D28	17
	D42	12
	Unscheduled days after D42	2
	Total number of AL- treated recurrent infections	44
DP	D21	0
	D28	3
	D42	14
	Unscheduled days after D42	3
	Total number of DP- treated recurrent infections	20

Parasitological data from the AL-treated patients revealed a 31.7% increase in parasite density in patients with a recurrent infection on day 21 compared to the corresponding baseline parasitaemia on day 0 (Table 2). As the corresponding parasite counts increased, there was a mean decline in the mean haemoglobin (Hb) levels on day 21 to 9.7 g/dL (Table 2). There was an 11.6% and 13.8% increase in Hb levels on day 28 and day 42 post-treatment in DP-treated patients, respectively, compared to a 9.4% and 7.2% increase on similar follow-up days in the AL-treated patients (Table 3).

**Table 2:** The parasite counts, haemoglobin level, and age of patients with recurrent parasitaemia on day 21, day 28, day 42 and on unscheduled days following artemisinin-lumefantrine (AL) treatment

Drug: AL	Samples with recurrent parasitaemia on D21		Samples with recurrent parasitaemia on D28		Samples with recurrent parasitaemia on D42		Samples with recurrent parasitaemia on unscheduled days	
	Counts on D0	Counts on D21	Counts on D0	Counts on D28	Counts on D0	Counts on D42	Counts on D0	Counts on unscheduled days
Parasite counts/µl Geometric mean	10032	13210	19696	5764	15697	3793	50689	20077
Haemoglobin, g/dL Mean (Hb)	10.7 ± 1.5	9.7 ± 1.9	10.6 ± 1.8	11.6 ± 1.8	11.0 ± 1.7	11.8 ± 1.4	9.6 ± 1.1	9.9 ± 1.2
Age, years mean	3.0 ± 1.3		3.0 ± 1.1		4.0 ± 1.5		4.5 ± 1.7

**Table 3:** The parasite counts, haemoglobin level, and age of patients with recurrent parasitaemia on day 28, day 42 and on unscheduled days following dihydroartemisinin-piperaquine (DP) treatment

Drug: DP	Samples with recurrent parasitaemia on D28		Samples with recurrent parasitaemia on D42		Samples with recurrent parasitaemia on unscheduled days	
	Counts on D0	Counts on D28	Counts on D0	Counts on D42	Counts on D0	Counts on unscheduled days
Parasite counts/µl Geometric mean	40962	3732	22011	6226	6428	22063
Haemoglobin, g/dL Mean (Hb)	9.5 ± 1.7	10.6 ± 1.9	9.4 ± 1.8	10.7 ± 1.9	9.6 ± 1.6	11.7 ± 1.1
Age, years, mean	2.75 ± 1.5		2.0 ± 1.2		2.6 ± 1.3	

### Analysis of recrudescent infections

**Fig. 1:**
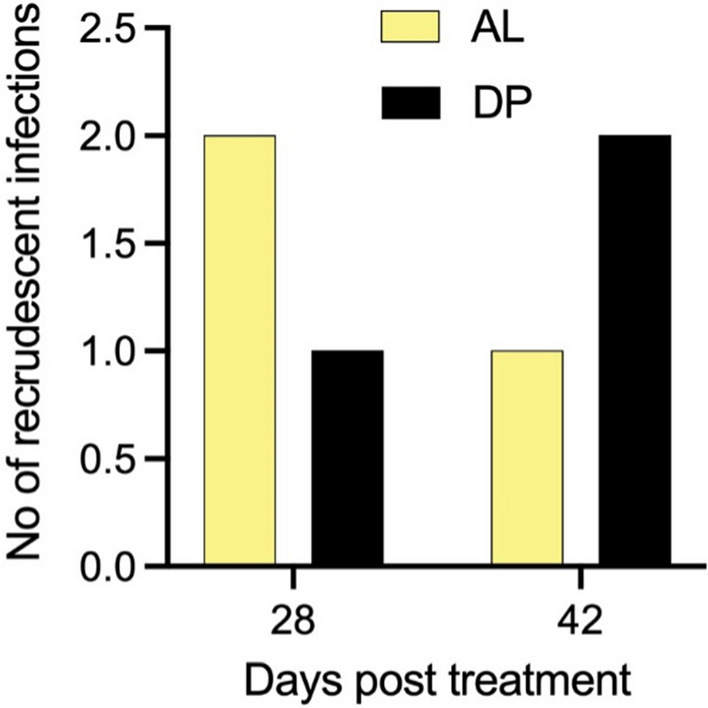
Number of recrudescence infections on post treatment follow up days after treatment with artemether-lumefantrine (AL) and dihydroartemisinin-piperaquine (DP)

Recrudescent infections were categorized as infections in which the alleles in paired samples were identical at day 0 and the day of recurrent infection (Additional file 1: Fig S1). Two recrudescent infections were detected on day 28 post-treatment and one recrudescent infection on day 42 post treatment in the AL treatment arm (Fig. 1). In the DP treatment arm, one recrudescent infection was detected on day 28 post-treatment and two recrudescent infections on day 42 post-treatment (Fig. 1). No recrudescence was detected on day 21 post-treatment and on unscheduled follow up days in both treatment arms.

### Analysis of the *Pfnfs1* gene in recurrent infections after AL-treatment

**Fig. 2:**
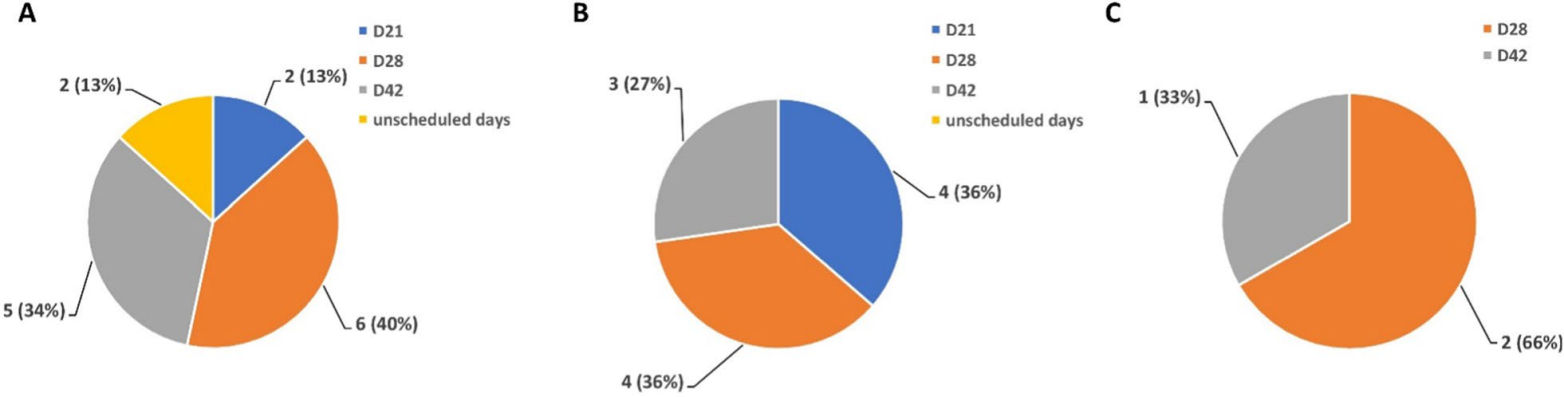
Number and percentage (%) frequency of **A** K65 wild-type allele in recurrent infections **B** K65Q mutant allele in recurrent infections and **C** K65 wild-type allele in recrudescence samples on day 21, day 28, day 42, and unscheduled days following treatment with artemether-lumefantrine (AL)

PCR amplification of the target region revealed a 686 bp fragment (Additional file 2: Fig S2). After sequencing of the *Pfnfs1* gene target region, 26 of the 44 AL-treated recurrent samples were confidently scored and analysed. 15 AL-treated recurrent samples possessed the K65 wild-type allele while the K65Q mutant allele was detected in 11 samples. The K65 wild-type allele was detected in two samples collected on day 21, six samples collected on day 28 and in five samples collected on day 42 (Fig. 2A). The K65Q mutant allele was detected in four samples on both day 21 and day 28 post treatment and in three samples collected on day 42 post treatment (Fig. 2B). The three recrudescent samples detected in the AL-treatment arm of the study all contained the K65 wild-type allele- two were detected on day 28 post treatment and one on day 42 post treatment (Fig. 2C).

### Analysis of the *Pfnfs1* gene in recurrent infections after DP-treatment

**Fig. 3:**
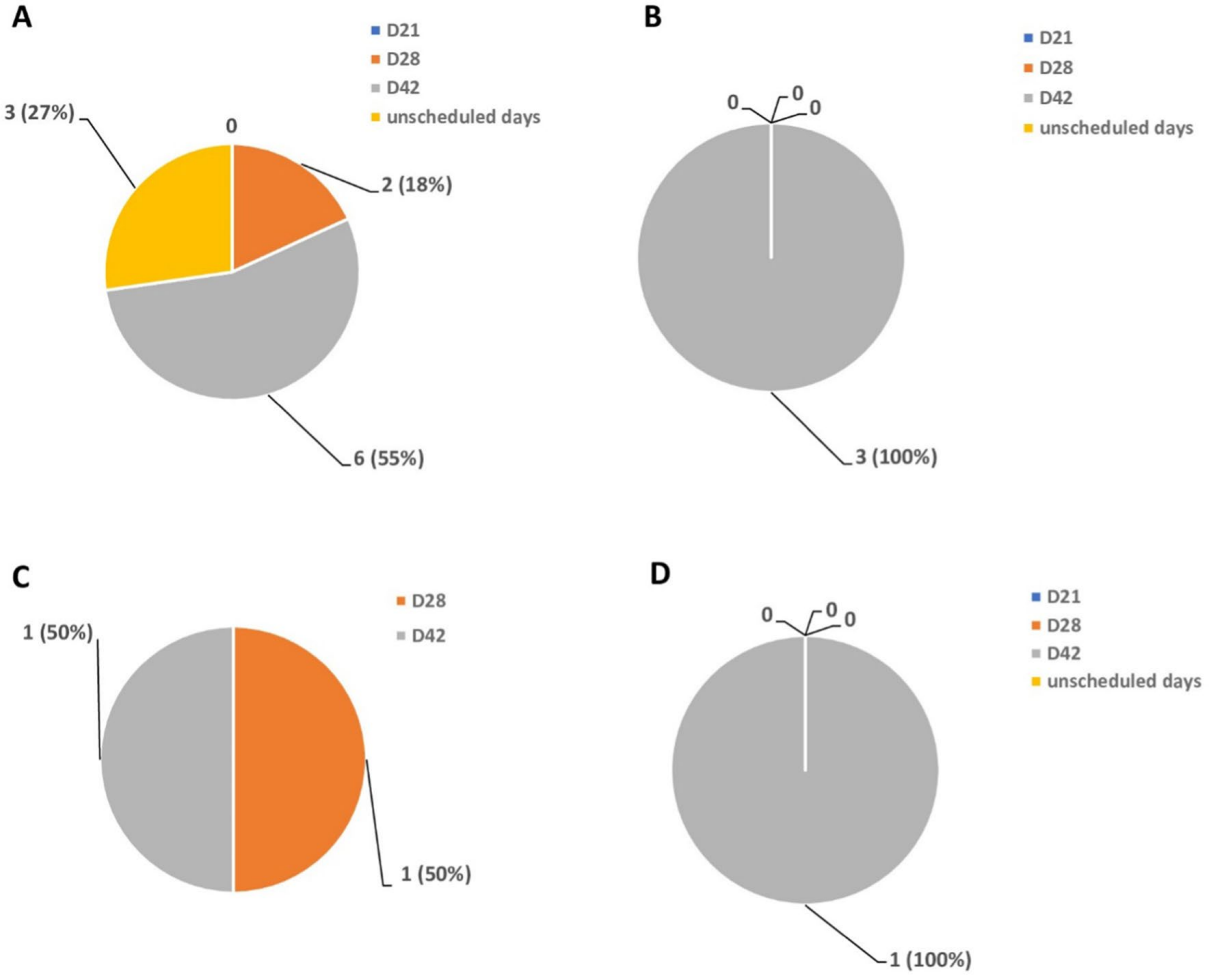
Number and percentage (%) frequency of **A** K65 wild-type allele in recurrent infections **B** K65Q mutant allele in recurrent infections **C** K65 wild-type allele in recrudescence infections **D** K65Q mutant allele in recrudescent infections on day 21, day 28, day 42, and unscheduled days following treatment with dihydroartemisinin-piperaquine (DP)

An evaluation of recurrent infections in the DP arm of the study revealed that 14 of the 20 DP-treated samples were confidently scored and analysed after sequencing. 11 samples contained the K65 wild-type allele while three samples carried the K65Q mutant allele. The K65 wild-type allele typically associated with lumefantrine resistance was detected at a frequency of 18% on day 28. Interestingly, the highest frequency of the K65 wild type allele in both treatment arms was detected on day 42 post DP treatment at a frequency of 55% (Fig. 3A). Further probing of the recurrent samples showed that the K65Q mutant allele was only detected on day 42 post-treatment (Fig. 3B). Two of the three recrudescent samples detected in the DP-treatment arm of the study contained the K65 wild type allele- one was detected on day 28 post treatment and the other on day 42 post-treatment (Fig. 3C). One recrudescent sample collected on day 42 post-treatment contained the K65Q mutant allele (Fig. 3D).

## Discussion

In the AL treated patients, the increase in parasite counts is hypothesized to be consistent with expected declining LM concentrations below the minimum inhibitory concentration threshold. Studies have shown that an increase in the prevalence and intensity of malaria-induced anaemia has been associated with recurrent malaria infections caused by parasite recrudescence [33]. The high parasite counts on day 21 are presumed to result in the depletion of circulating erythrocytes contributing to low Hb levels and malaria-induced anaemia. The rapid depletion of red blood cells and inflammation have been associated with lower erythrocyte recovery in malaria infections [34]. Parasitaemia was continually reported in consequent follow-up days after the first detection of recurrent parasitaemia in both AL and DP-treated patients. Previous studies have suggested parasite dormancy or quiescence causes delayed parasite clearance [35, 36]. A higher haemoglobin recovery was reported in the DP-treated patients compared to the AL-treated patients. In Uganda, DP was also reported to have a better haemoglobin recovery, as shown by the higher mean increase in Hb levels compared to AL [37]. In most cases however, Hb levels have been reported to recover and increase despite treatment failure [38]. Treatment failure after DP is rare and has only been registered in three countries in Africa, and these studies also showed treatment failure with AL [39–41].

Overall, 9% of total recurrent infections were recrudescent infections while 91% were new infections. The high number of new infections is consistent with high malaria transmission in the study site. In these infections, proportions of the K65 wild-type allele and the K65Q mutant allele could be an indicator of their distribution in the parasites circulating in the region. In this study, the rate of treatment failure was 1.80% in the AL arm of the study and 1.78% in the DP arm of the study and underscores the exigency for consistent monitoring of anti-malarial drug efficacy in field settings.

An increase in the K65 wild-type allele has been shown to increase with AL use and the IC_50_ of LM against *P. falciparum* field isolates [24]. Additionally, a mathematical model predicted an expansion of acquired resistance to AL between day 20 and 39 post-treatment [42]. In this study the parasite samples were not collected for subsequent in vitro cultures and the determination of the IC_50_ values. It was observed that recurrent parasitaemia emerged earlier in AL than in DP-treated patients (Table 1). The elimination half-life of lumefantrine is approximately four to five days [43], while that of piperaquine has been estimated to be between 23 to 33 days [44]. Therefore, the difference in the onset of recurrent parasitaemia between the two treatments may be due to the difference in drug plasma elimination rates in the patients. Moreover, a 56% higher incidence of recurrent infection following treatment with AL than treatment with DP was observed, which may point toward parasites adapting to AL drug pressure compared to DP. There is a reciprocal resistance between aryl alcohol drugs, such as LM, and aminoquinoline drugs, such as chloroquine (CQ) and PQ [45]. The selection of K65 may, therefore, confer higher susceptibility to aminoquinoline drugs, thus delayed parasite recurrence. There was a higher frequency of the K65 wild type allele in this study compared to a study conducted in Kilifi, a coastal region of Kenya [46]. This could be attributed to the difference in malaria transmission between the two regions. The coastal region experiences moderate malaria transmission while the Lake endemic region where this study was conducted is under high and stable malaria transmission.

In this study, recurrent infections were reported up to day 42 post AL treatment. Furthermore, the K65 wild-type allele was detected in the recrudescent sample collected on day 42 post-infection. The detection of an increased number of K65 wild-type alleles in recurrent infections from day 21 to day 42 of the follow-up period may suggest a possible direction for selection following continued AL treatment within the region. Collectively, these data underscore the benefit of a 42-day minimum follow-up period in malaria drug efficacy studies and surveillance of parasite drug resistance. Treatment failure after AL treatment may be caused by poor drug absorption and bioavailability, patient noncompliance with drug dosages, and parasite resistance. Treatment failure after AL therapy has been reported in Angola [47], the Democratic Republic of Congo [40] and Burkina Faso [41]. A recent study in Busia district of Eastern Uganda, which is in relatively close proximity to the site assessed in this study, also reported high malaria prevalence and low efficacy of AL [39]. Treatment failures have also been reported in travelers from Africa [48, 49]. These studies suggest a consistent trend of AL failure and subsequent parasite recrudescence. Although poor LM absorption is not entirely ruled out as a likely contributor to recurrent parasitaemia observed in this study, detecting K65 mutation in the AL arm strongly suggests that recurrent infections may be highly linked to the K65 mutation rather than poor absorption.

Recurrent parasitaemia in the DP arm was detected from day 28, after which an increase in the number of recurrent infections with time culminated in peak recurrent infections on day 42 post-treatment (Table 1). The highest number of recrudescent infections were also detected on day 42 post treatment. It is now well-established that artemisinin derivatives induce dormant parasites that fuel recurrent infection associated with parasite recrudescence in vitro and in vivo [50–53]. The persistent parasites reemerge following drug treatment when the drug concentration is subtherapeutic [54]. This may imply that the slow PQ elimination profile may delay recrudescent parasites and remotely prevent new infections. However, this observation is not unique; a clinical study in Uganda also reported increased recurrent parasitaemia linked to recrudescence in DP-treated individuals from day 28 to day 42 [39]. Studies in Asia have also reported recrudescent infections after DP treatment starting from day 28 of the follow-up period [55, 56]. Recrudescence was also reported in patients treated with DP more than six weeks post-treatment [57]. The relatively longer elimination half-life of piperaquine may result in a protracted advantage in suppressing parasite recurrence hence the onset of recurrent parasitaemia on day 28 in this study. After this period, the drug's minimum inhibitory concentration diminishes, and residual parasites not yet cleared may expand, increasing the probability of recurrent infections. Furthermore, due to the long elimination profile of PQ, dwindling plasma drug concentrations could result in the selection and propagation of parasites resistant to the drug.

## Conclusion

The iron-sulfur cluster synthesis (ISCS) pathway has been proposed as a promising target for anti-malarial drugs, and *Pfnfs1* is critical in driving the pathway. Using samples collected after more than ten years of ACT use in Kenya, the data generated shows the presence of the K65 lumefantrine resistance selection marker in recurrent infections in Matayos, Western Kenya. Due to the threat of emerging drug-resistant *P. falciparum* parasites, there is a need for continued monitoring of molecular markers of ACT treatment failure to complement clinical data in informing treatment policy in Kenya and other high malaria transmission areas. Future studies should also evaluate the drug susceptibility profile of the recrudescent parasites and the association of observed mutations with drug susceptibility.

## Supplementary Information

**Additional file 1: Fig S1:** Gel image of PCR products from amplification of the *Plasmodium falciparum* msp2and *Plasmodium falciparum* msp1gene from selected patients isolates. Lane 1- 100bp ladder; Lane 2- Patient sample 1, Day 0, MSP2 Lane 3- Patient sample 1, Day 28, MSP2, Lane 4- Patient sample 1, Day 0, MSP1, Lane 5- Patient sample 1, Day 28, MSP1; Lane 6- 100bp ladder, Lane 7- Patient sample 2, Day 0, MSP2, Lane 8- Patient sample 2, Day 42, MSP2, Lane 9- Patient sample 2, Day 0, MSP1, Lane 10- Patient sample 2, Day 42, MSP1; Lane 11- 100bp ladder, Lane 12- Patient sample 3, Day 0, MSP2, Lane 13- Patient sample 3, Day 21, MSP2, Lane 14- Patient sample 3, Day 0, MSP1, Lane 15- Patient sample 3, Day 21, MSP1, Lane 16- 100bp ladder. Lane 2-5: New infections, Lane 7-10: Recrudescent infections, Lane 12-15: New infection.

**Additional file 2: Fig S2**: Gel image of PCR products from amplification of the *Plasmodium falciparum* cysteine desulfurase IscSgene from selected patients isolates. Lane 1- 100bp ladder**, **Lane 2- Patient sample 1**, **Lane 3- Patient sample 2**, **Lane 4- Patient sample 3**, **Lane 5- Patient sample 4**, **Lane 6- Patient sample 5**, **Lane 7- Patient sample 6**, **Lane 8- Patient sample 7**, **Lane 9- Patient sample 8**, **Lane 10- Patient sample 9**, **Lane 11- Patient sample 10**, **Lane 12- Patient sample 11**, **Lane 13- Patient sample 12**, **Lane 14- Patient sample 13**, **Lane 15- Patient sample 14**, **Lane 16- 100bp ladder.

## Data Availability

The data set used in this study is available and can be shared upon reasonable request to the corresponding author.
