# Supplementary figures and images for: Prevalence of mutations in the cysteine desulfurase IscS (Pfnfs1) gene in recurrent Plasmodium falciparum infections following artemether-lumefantrine (AL) and dihydroartemisinin-piperaquine (DP) treatment in Matayos, Western Kenya
Source: Malar J. 2023 May 19;22:158. doi: 10.1186/s12936-023-04587-2 (PMC10197370; doi:10.1186/s12936-023-04587-2)

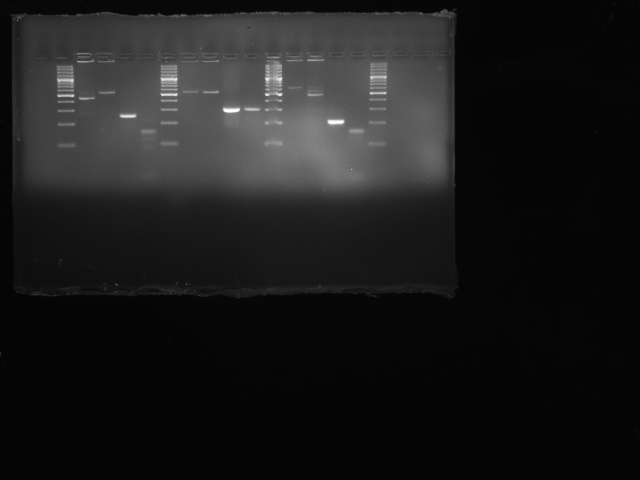

Supplement: Supplementary file 1 — Additional file 1: Fig S1: Gel image of PCR products from amplification of the Plasmodium falciparum msp2and Plasmodium falciparum msp1gene from selected patients isolates. Lane 1- 100bp ladder; Lane 2- Patient sample 1, Day 0, MSP2 Lane 3- Patient sample 1, Day 28, MSP2, Lane 4- Patient sample 1, Day 0, MSP1, Lane 5- Patient sample 1, Day 28, MSP1; Lane 6- 100bp ladder, Lane 7- Patient sample 2, Day 0, MSP2, Lane 8- Patient sample 2, Day 42, MSP2, Lane 9- Patient sample 2, Day 0, MSP1, Lane 10- Patient sample 2, Day 42, MSP1; Lane 11- 100bp ladder, Lane 12- Patient sample 3, Day 0, MSP2, Lane 13- Patient sample 3, Day 21, MSP2, Lane 14- Patient sample 3, Day 0, MSP1, Lane 15- Patient sample 3, Day 21, MSP1, Lane 16- 100bp ladder. Lane 2-5: New infections, Lane 7-10: Recrudescent infections, Lane 12-15: New infection. [file 12936_2023_4587_MOESM1_ESM.tif]

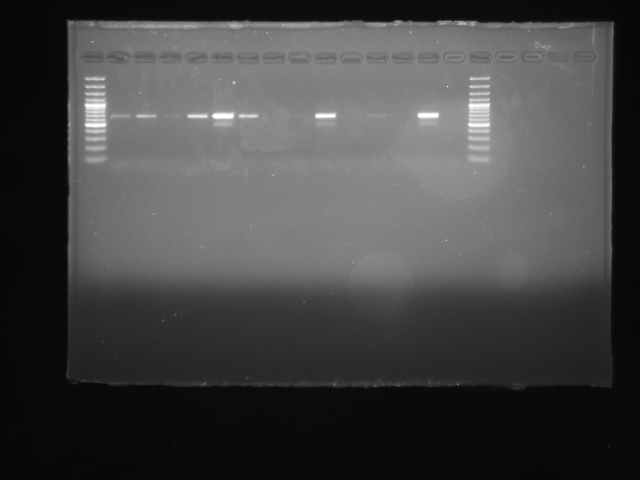

Supplement: Supplementary file 2 — Additional file 2: Fig S2: Gel image of PCR products from amplification of the Plasmodium falciparum cysteine desulfurase IscSgene from selected patients isolates. Lane 1- 100bp ladder, Lane 2- Patient sample 1, Lane 3- Patient sample 2, Lane 4- Patient sample 3, Lane 5- Patient sample 4, Lane 6- Patient sample 5, Lane 7- Patient sample 6, Lane 8- Patient sample 7, Lane 9- Patient sample 8, Lane 10- Patient sample 9, Lane 11- Patient sample 10, Lane 12- Patient sample 11, Lane 13- Patient sample 12, Lane 14- Patient sample 13, Lane 15- Patient sample 14, Lane 16- 100bp ladder. [file 12936_2023_4587_MOESM2_ESM.tif]
